# Electro-Oculography and Proprioceptive Calibration Enable Horizontal and Vertical Gaze Estimation, Even with Eyes Closed

**DOI:** 10.3390/s25216754

**Published:** 2025-11-04

**Authors:** Xin Wei, Felix Dollack, Kiyoshi Kiyokawa, Monica Perusquía-Hernández

**Affiliations:** Graduate School of Science and Technology, Nara Institute of Science and Technology (NAIST), Ikoma 630-0192, Japan; wei.xin.wy0@is.naist.jp (X.W.); felix.d@is.naist.jp (F.D.); kiyo@is.naist.jp (K.K.)

**Keywords:** electro-oculography, signal processing, eyes closed, gaze direction estimation

## Abstract

Eye movement is an important tool used to investigate cognition. It also serves as input in human–computer interfaces for assistive technology. It can be measured with camera-based eye tracking and electro-oculography (EOG). EOG does not rely on eye visibility and can be measured even when the eyes are closed. We investigated the feasibility of detecting the gaze direction using EOG while having the eyes closed. A total of 15 participants performed a proprioceptive calibration task with open and closed eyes, while their eye movement was recorded with a camera-based eye tracker and with EOG. The calibration was guided by the participants’ hand motions following a pattern of felt dots on cardboard. Our cross-correlation analysis revealed reliable temporal synchronization between gaze-related signals and the instructed trajectory across all conditions. Statistical comparison tests and equivalence tests demonstrated that EOG tracking was statistically equivalent to the camera-based eye tracker gaze direction during the eyes-open condition. The camera-based eye-tracking glasses do not support tracking with closed eyes. Therefore, we evaluated the EOG-based gaze estimates during the eyes-closed trials by comparing them to the instructed trajectory. The results showed that EOG signals, guided by proprioceptive cues, followed the instructed path and achieved a significantly greater accuracy than shuffled control data, which represented a chance-level performance. This demonstrates the advantage of EOG when camera-based eye tracking is infeasible, and it paves the way for the development of eye-movement input interfaces for blind people, research on eye movement direction when the eyes are closed, and the early detection of diseases.

## 1. Introduction

Eye tracking is the measurement of eye movements, or more specifically, the gaze direction and fixation patterns. On the one hand, the gaze direction can be controlled voluntarily. Therefore, it is used for human–computer interfaces where limb movement is not possible [1] or when a hands-free interaction is desirable [2]. On the other hand, involuntary eye movements are often correlated with cognitive processes in the human brain, such as attention and preferences [3,4].

Considering these two use cases, our focus was on the voluntarily controlled gaze direction because it is useful for gaze-based interaction designs for vulnerable populations such as blind people and locked-in patients [5]. For example, blind people could use their eyes as a joystick during audio- and tactile-guided navigation [6,7]. In particular, when the hands are used as a surface for tactile displays [8], eye movements can provide an additional input channel for navigation and object manipulation. In this case, blind people are not using their eyes for vision anymore; therefore, the eyes’ voluntary movements are free for interaction.

Gaze direction is typically represented in a two-dimensional view reference plane. Vergence cues for the eye direction are small, but have been used to assess the depth gaze in virtual and augmented reality displays or in non-display interactions [9,10,11]. However, when people are interacting with screens, the vergence between the eyes remains the same, presenting a challenge to identify the depth cues and to point at visual target interactions [12]. Furthermore, when using gaze as an interaction input with the eyes closed, the depth cues are unavailable. Therefore, we focused on gaze estimation in a two-dimensional plane.

There are different techniques that can be used to measure the gaze direction, and each of them has its distinct advantages and shortcomings. One such technology is computer vision (CV), where an infrared light pattern is projected onto the pupil, and the reflection and changes therein are recorded with a camera [13]. This technique achieves good results, is easy to set up, and is not invasive. Some of the downsides of CV are the need to have uncovered access to the eyes and its sensitivity to illumination changes, as well as the reflection of other light sources like the infrared portion of sunlight or reflections on glasses [14,15,16,17]. When the eye area is partially or intermittently covered by eyelids, hair, or external accessories, the accuracy and reliability of CV-based eye tracking can be significantly compromised. This is particularly problematic for users with certain eye conditions that cause partial eyelid closure or obstruction of the eye region, such as ptosis (drooping eyelids) or habitual squinting resulting from poor visual acuity [18,19,20]. Additionally, image processing in CV-based systems often requires a high amount of computational resources. Recent research has sought to mitigate this demand by introducing techniques such as feature maps similarity knowledge transfer, which enables accurate gaze estimations from low-resolution images [21]. Another line of work improved the computational efficiency by suppressing redundant facial information and enhancing the extraction of gaze-relevant features through attention-based mechanisms [22]. An alternative method involves the use of search coils [23]. Contact lenses with a very small metal coil are placed onto the eyes and measured within an electromagnetic field. The movement of the search coils leads to changes in the magnetic field that are measurable at a very high precision, even with closed eyes, but the wire inside the eye leads to some discomfort and can, therefore, only be used for up to 30 min as recommended by the manufacturers [24]. Electro-oculography (EOG) is a third technique that is less invasive and can, as we will show, measure the gaze direction with closed eyes. The electrical difference between the cornea at the front of the eye and the retina at the back of the eye can be measured with electrodes pasted horizontally and vertically outside of the eyes. However, the accuracy of EOG is often limited by noise resulting from the overlap with other biological signals, such as facial electromyography (EMG). Additionally, the basic charge of the eye is different depending on the lighting situation, as the eye’s potential decreases in darkness, reaching a dark trough after 8–12 min, and rises after the lights are turned on again, peaking about 10 min later [25]. Therefore, lighting conditions might cause drifts in the EOG measurements as well.

Depending on the situation, it can still be worthwhile to choose EOG over CV, despite the challenges involved with EOG measurement. A summary comparison of EOG- and CV-based eye-tracking methods is provided in Table 1. The limitations of CV-based eye tracking—including the sensitivity to reflections, a poor performance with glasses, and failure under eye occlusion—make it unsuitable for certain scenarios where EOG can be used unrestrictedly. These scenarios where EOG is advantageous include sleep monitoring, use in high- or low-light environments, or use with participants wearing prescription lenses. However, most video-based and EOG-based eye-tracking techniques rely on visual fixation for calibration. Because of this, they are inherently unsuitable not only for closed-eye conditions, but also for individuals who are blind or partially sighted and cannot perceive visual targets even with their eyes open.

To address this critical limitation, we introduced a proprioceptive calibration method that uses a tactile cardboard interface with a pattern of felt dots. This task leverages the body’s internal sense of limb position to guide gaze alignment, thereby allowing users to perform guided fixations under closed-eye conditions to enable consistent and repeatable calibration without relying on visual input [30].

The main contributions of this study are as follows:

- We introduced a proprioceptive calibration method that enables EOG calibration in the absence of visual input.
- We propose a novel approach for estimating the gaze direction using EOG signals when the eyes are closed.
- We established eye tracking as a benchmark for evaluating EOG-based gaze direction estimation by comparing both modalities under eyes-open conditions.
- We validated the effectiveness of EOG-based gaze estimation under eyes-closed conditions by comparing aligned EOG trajectory data with shuffled (chance-level) control data.

Estimating the gaze direction with closed eyes would be beneficial when designing supportive technology for groups dependent on human–computer interaction for an improved well-being and quality of life, such as people with locked-in syndrome [5] or amyotrophic lateral sclerosis [1]. Furthermore, it would allow experiments with blind participants and the investigation of cognition through eye tracking, where retinal inputs are unavailable—for example, in the case of audio-guided navigation [31]. EOG has also been widely used in sleep monitoring, particularly within polysomnography (PSG), where it supports sleep staging by capturing gross eye movements [32]. However, several studies in sleep and dream research have gone further by analyzing the characteristics and directions of eye movements during REM sleep, and our method offers a potential tool to support such advanced investigations. For instance, previous work has shown alignment between EOG-based eye movement directions and dream reports [33], demonstrated that smooth-pursuit tracking during REM sleep more closely resembles perception than imagination [34], and revealed that REM gaze shifts in mice are linked to shifts in the virtual head direction [35].

The remainder of this paper is organized as follows: The Section 2 reviews prior research on gaze estimation, beginning with EOG-based approaches under eyes-open conditions, followed by techniques developed for eyes-closed scenarios. The Section 3 outlines the experimental details, including participant recruitment, the experimental design, the apparatus, and the data-collection procedures. The Section 4 details the analytical methods used in this study, including the signal preprocessing pipeline and evaluation strategies. The Section 5 presents the experimental findings. The Section 6 interprets the results in the context of the existing literature. Finally, the Section 7 outlines the current constraints of the study and proposes directions for future research.

## 2. Related Works

Gaze estimation has been widely used in vision science and HCI, including a wide range of applications, such as attention analyses, intention recognition, and hands-free interaction control. Among various gaze estimation methods, EOG with open eyes is a common input in the field of HCI to control wheelchairs, play games, and write text [36,37,38]. It is also employed for recognizing user activities such as reading, writing, or watching videos [39]. Several algorithms have been proposed to estimate gaze direction using EOG signals with open eyes [40,41,42,43]. These methods typically require calibration to determine the direction and amplitude of eye movements. This calibration process often involves associating EOG signal amplitudes with known gaze targets to derive user-specific transfer functions, based on the well-established linear relationship between the EOG amplitude and the visual angle of gaze shifts [37,39,44]. For example, Yan et al. adopted a calibration procedure in which participants were guided to fixate on 24 predefined gaze targets positioned at angular offsets of 30°, 45°, 60°, and 75° to the left and right, across three vertical levels: up, middle, and down [45]. Manabe et al. proposed a horizontal gaze estimation approach using nine predefined gaze targets ranging from −40° to +40° from left to right with an interval of 10° [46]. Instead of using a predefined calibration grid, Barbara et al. employed a biophysical battery model of the eye and estimated the model parameters using EOG signals recorded while the participants fixated on targets that appeared randomly on the screen [47]. However, these calibrations require open eyes to fixate on predefined screen-based targets, limiting their applicability in closed-eye scenarios.

In 1980, the gaze direction or eye-in-head position was estimated based on a maximum movement calibration during experiments to investigate eye movements in the blind [48]. Hsieh et al. investigated the relationship between EOG signals and visual features of closed-eye motion to synthesize artificial EOG signals using computer vision for non-invasive sleep tracking [49]. Recent advances have explored various modalities and applications for gaze estimation and interaction under closed-eye conditions. Findling et al. introduced a camera-based recognition of closed-eye gaze gestures using optical flow from eye-facing cameras in smart glasses [50]. They proposed four closed-eye gaze gesture protocols and a processing pipeline for classifying eyelid movement patterns. Extending this work, the authors applied EOG to enable closed-eye gaze gesture input for secure mobile authentication [51]. They designed a nine-character EOG-based gesture alphabet and used sensors embedded in the nose pads and bridge of smart glasses to enable gaze gesture detection for password entry. Tamaki et al. also explored EOG-based closed-eye interactions for users with severe motor impairments, achieving the reliable detection of upward eye movements through a combination of thresholding and k-nearest neighbor classification methods [52]. These gesture-based studies demonstrate that EOG can operate under eyes-closed conditions, but they did not attempt to estimate the continuous gaze direction or analyze fine-grained gaze movements. Beyond interaction systems, Ben Barak-Dror et al. demonstrated a touchless short-wave infrared (SWIR) imaging method capable of tracking pupil dynamics and estimating the gaze direction through closed eyelids [53]. This approach uses a U-NET architecture to reconstruct an open-eye model from closed-eye SWIR images, thereby enabling the estimation of pupil size and gaze shifts with a sub-second temporal resolution. To validate the accuracy of gaze estimation, the study employed a protocol in which the participants kept one eye open while manually holding the other eye closed using a finger, allowing for a comparison between the estimated gaze and simultaneous open-eye measurements used as the ground truth, which relies on the assumption of conjugate eye movement between both eyes. However, this assumption does not hold universally; for instance, it does not hold in an individual with strabismus. Moreover, physically holding one eye closed using a finger may restrict natural ocular motion, potentially compromising the validity of the recorded eye movement data. Additionally, the need for active illumination and computational resources makes visual-based methods less suitable for long-term applications such as sleep monitoring, dream research, or critical care settings, where low-power and unobtrusive methods are preferred. MacNeil et al. proposed a calibrated EOG method that aligns EOG signals with ground-truth data from a pupil–corneal reflection tracker to enable the measurement of closed-eye movement kinematics [54,55]. The calibration factor was estimated under open-eye, normal-illumination conditions and then applied to adjust the EOG signals recorded in darkness and during tasks performed with eyes closed. Although this study demonstrates the feasibility of closed-eye movement tracking using calibrated EOG, it focused solely on horizontal eye movements due to a poor signal quality in the vertical EOG channel. In addition, MacNeil et al.’s memory-based design assumes that participants can visually perceive and memorize the calibration pattern, which is not feasible for blind individuals, and may introduce additional sources of error from relying on mental imagery and saccadic execution without visual feedback. To address these limitations, we propose a tactile-guided proprioceptive calibration method that uses a pattern of felt dots on cardboard to guide the gaze with eyes open or closed, under both illuminated and dark conditions. This method does not require visual fixation and provides tactile spatial references that are more reliable than memory-based calibration. In addition, we explored both horizontal and vertical eye movements in this work. In summary, our method differs from prior work in several key aspects: (1) it is grounded in physiological signals and does not depend on visual access; (2) it supports both horizontal and vertical gaze estimation; and (3) it is inherently compatible with individuals who are blind or partially sighted, as it does not require sighted calibration or visual stimuli at any stage of the procedure.

## 3. Materials and Methods

### 3.1. Participants

Five volunteers (three female, mean age: 25.6 years old, SD = 2.7) participated in a pilot study. The pilot study was conducted as an initial test of the experimental procedure and apparatus. Based on its outcome, the main experiment was then carried out with the finalized procedure using paid participants. During the pilot study, there were procedural changes, and we experienced technical difficulties with the eye tracker. Only the data for the last participant used the same procedure as the main experiment and were kept for further analysis. For the main experiment, ten paid participants (five female, mean age: 28 years old, SD = 9.8) were recruited. One participant completed half of the trials due to limited time, performing only the lights-off condition. Therefore, there were 11 participants whose data were analyzed. The study was conducted in accordance with the Declaration of Helsinki. The experimental procedure was explained to the participants beforehand, and all gave informed consent to participate.

### 3.2. Experiment Design

**Figure 1:**
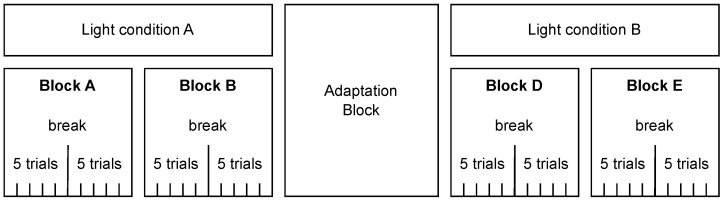
Block diagram of the experimental design. Each participant completed four blocks of trials (Blocks A–B under light condition A and Blocks D–E under light condition B), with the order of light conditions and blocks randomized. Light conditions A and B could be lights on or off. Each block consisted of ten trials, with a short break after five trials. There was an adaptation phase with lights off and eyes closed between the two light conditions.

We used a 2×2 within-subjects design with the factors *light* (on/off) and *eyes* (open/closed). Each participant completed four blocks (two per light condition; see Figure 1); the order of light conditions and blocks was randomized. Each block comprised 10 trials with the eye state (open/closed) randomized across blocks. The participants took a short break after every fifth trial. Between the two light conditions, an adaptation phase with lights off was inserted; the participants kept their eyes closed and listened to an audiobook read in English.

### 3.3. Apparatus

**Figure 2:**
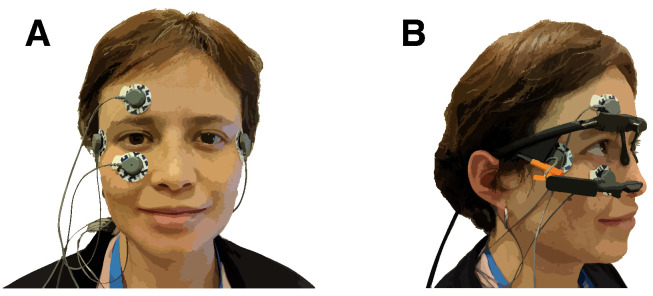
(**A**) Electrode placement. Electrodes above and below the right eye were used to collect vertical eye movement. Electrodes below the temples at the lateral side of the eyes recorded horizontal eye movement. A ground electrode was attached to the right mastoid bone. (**B**) The eye tracker was placed above the electrodes.

The experiment took place in a soundproof booth. The participants were seated at a table with their heads on a chin rest. In front of the participants, at a distance of 28 cm, was the target pattern on a cardboard square taped to a screen. The target pattern used in the experiment was made from round felt stickers. The participants wore active noise-canceling headphones (ATH-ANC7b-SViS, Audio-Technica, Tokyo, Japan) to hear the instructions and suppress environmental noise. The participants also wore eye-tracking glasses (Pupil Core, Pupil Labs, Berlin, Germany) with a world-facing camera and monocular tracking of the right eye [56]. The eye-tracking glasses were connected to a MacBook Air (3.1 GHz Intel Core i7, 16 GB RAM, 2017, macOS High Sierra, Apple, Cupertino, CA, USA) running Pupil Capture (version 1.13.31, Pupil Labs, Berlin, Germany). The experiment was controlled by custom software written in C++11 with openFrameworks 0.9.4 [57]. Eye movement was recorded using electro-oculography (EOG). The participants wore disposable electrodes (Kendall ARBO H124SG, CardinalHealth, Dublin, OH, USA) above and below their right eye (vertical direction) and outside their right and left eye (horizontal direction), as shown in Figure 2. A fifth electrode on the mastoid behind the right ear served as a reference. The electrodes were connected to a Shimmer3 ECG/EMG Bluetooth device (Shimmer, Dublin, Ireland) that was placed into a pouch strapped diagonally across the participants’ backs.

An Arduino microcontroller (Arduino SA, Monza, Italy) connected with USB 2.0 to the MacBook Air received synchronization signals from the custom software through a serial port. A Shimmer3 Expansion device was connected to the Arduino with a 3.5 mm audio-jack cable and recorded the synchronization signals. A 5 V signal presented on the audio-jack cable was interrupted for a duration of 50 ms after receiving a synchronization signal. The shimmer devices recorded to internal memory and streamed to a Lenovo Ideapad 330S 15.6 (Lenovo, Beijing, China). The Shimmer ConsensysPRO v1.5.0 was used to synchronize the recordings from both Shimmer devices and export the data for further processing.

### 3.4. Procedure

**Figure 3:**
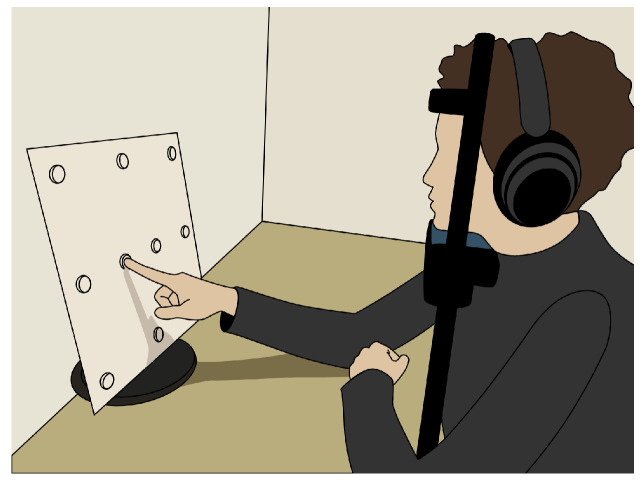
Sketch of the measurement setup. The participants were seated at a table with the target pattern in front of them. The head was stabilized by a chin rest, and the participants wore headphones.

**Figure 4:**
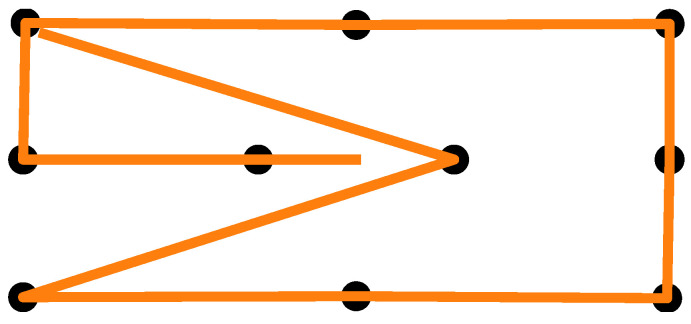
Calibration pattern used as a guide for the eye movement.

After giving consent to participate in the study, the participants’ skin above and below the right eye, as well as at the corner of both eyes and behind the right ear, was cleaned with alcohol cleaning wipes. EOG electrodes were placed on the cleaned skin areas and connected to a Shimmer unit that was stored in a small bag over the participant’s shoulder. A quick visual signal check was performed to confirm the electrode placement by means of the signal quality. Next, the participants were given the eye-tracking glasses, and the experimenter adjusted the eye camera. The participants were asked to use a headrest that was mounted on a table in front of them to keep their head in a stable position. At a distance of 28 cm in front of the headrest was a widescreen monitor. Using the Tobii Glasses PyController 2.2.4 [58], the eye tracker was calibrated. After successful calibration, a cardboard featuring felt-dot tactile markers (raised dots as shown in Figure 3) was pasted on top of the monitor. The participants completed two to three practice trials to familiarize themselves with the tactile pattern and the auditory signals. The experiment employed a 2 × 2 within-subjects factorial design with the lighting condition (lights on vs. lights off) and eye state (eyes open vs. eyes closed) as factors, resulting in four experimental conditions. The order of the lighting conditions was counterbalanced across participants, and within each lighting condition, the order of eye states was also counterbalanced. The participants performed two blocks in each lighting condition with a short one-minute break after five trials and in between the blocks. A block consisted of 10 measurements in total. For all four experimental conditions, the participants were instructed to touch the markers on the cardboard with their index finger and direct their gaze toward the tip of their finger. When the participants heard an instructional beep at 250 Hz, they had to move their finger between the markers while maintaining real or imaginary eye contact with the tip of their finger, depending on the experiment condition. The marker pattern is shown in Figure 4. Each trial began with a two-second initial phase, during which the participants fixated on the center of the cardboard. Afterward, they sequentially fixated on the 10 markers shown in Figure 4, with each fixation lasting two seconds. The upper-left marker was used twice, resulting in a total of 11 fixations. Combined with the initial phase, this yielded a trial duration of 24 s. After the first lighting condition, the lights in the room were turned off, or kept off, and the participants closed their eyes for 15 min while listening to an English audiobook read in a male voice. This adaptation phase was intended to mitigate the signal drift due to dilating pupils during lighting changes, but was used for all condition combinations. After the adaptation phase, the participants performed two more blocks in the other lighting condition—again, with a one-minute break in between the blocks. After the experiment, the participants removed the eye-tracking glasses and the experimenter disconnected the Shimmer unit and assisted the participant in removing the electrodes.

## 4. Analysis

### 4.1. Processing

**Figure 5:**
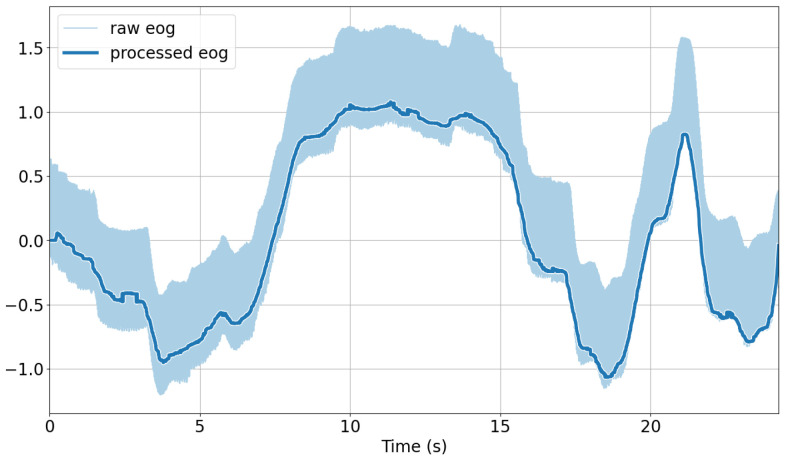
Comparison of raw and preprocessed EOG signals over time.

**EOG**: The signals were linear-detrended using the detrend function of the SciPy Python library [59]. A median filter with a window length of 200 ms was applied to the detrended signals using SciPy’s medfilt function. Finally, for the removal of eye blinks from the vertical signal component, we applied the algorithm devised by Bulling et al. [39] to identify blinks and interpolate linearly. The continuous 1D wavelet coefficients at a scale of 20 using a Haar mother wavelet were computed. A blink was characterized by a large positive peak directly followed by a negative peak in the resulting coefficient vector. Peaks were detected if their magnitude was outside of the average signal plus one standard deviation. The time between the two peaks of a blink is typically less than 0.5 s, which was chosen as the threshold to determine the interval between peaks as a blink. The identified blinks were then linearly interpolated across each interval using five samples on either side to replace the blink segment. For analysis purposes, the standard deviation using a sliding window approach with a window length of 31 ms was extracted. An illustration of the raw versus preprocessed EOG signals after applying these steps is shown in Figure 5.

**CV**: The recordings of the eye-tracking glasses were filtered using a Hampel filter to remove signal components that deviated 3σ from the standard deviation in a sliding window with a length of 100 ms. Finally, a median filter with a window length of 100 ms was applied.

The mean of both the EOG and the eye-tracking signals was subtracted before they were transformed to z-scores.

### 4.2. Comparison of EOG and Eye-Tracking Similarity Between Eyes-Open Lights-On and Lights-Off Conditions

To assess the similarity between the EOG and eye-tracking signals under different lighting conditions during eyes-open trials, we computed the participant-wise grand-average time series for each modality and condition. Specifically, we computed the grand-average signals by averaging the signals across the 10 trials associated with each lighting condition (i.e., EOG in lights-on/off and eye tracking in lights-on/off). One participant who did not complete the lights-on condition was excluded from this comparison. To quantify the alignment between EOG and eye tracking, we computed the cosine similarity between paired signals: (1) EOG vs. eye tracking under lights-on conditions, and (2) EOG vs. eye tracking under lights-off conditions. This was performed separately for the horizontal and vertical components.

### 4.3. Similarity Between Ground Truth, Eye Tracking, and EOG

To assess the temporal similarity between the physiological signals and the target trajectories, we computed the normalized cross-correlation for each participant and each pair of signals of interest. The analysis included comparisons between the EOG signals (recorded during eyes-open and eyes-closed trials), eye-tracking signals (during eyes-open trials), and the corresponding horizontal and vertical target trajectories. The cross-correlation was computed using full convolution, and the resulting coefficients and lags were normalized to the [0, 1] range. For this analysis, we used grand averages calculated by pooling the trials across both lighting conditions. The eyes-closed EOG signals were averaged over 20 eyes-closed trials, including both light conditions. As no eye-tracking data were available under the eyes-closed conditions, only the EOG signals were analyzed in that context. Similarly, the eyes-open EOG and eye-tracking signals were averaged over 20 eyes-open trials, including the two light conditions. One participant completed only half the experiment (10 trials per light condition); hence, the individual grand averages were based on either 10 or 20 trials. The group-level grand averages were calculated from a total of 210 trials. These averaged signals were used in subsequent statistical analyses.

### 4.4. Statistical Comparisons

All the statistical analyses were conducted in Python 3.8.18, utilizing the SciPy (v1.10.1) and Statsmodels (v0.14.1) libraries.

#### 4.4.1. Comparing EOG and Eye-Tracking Signals for the Eyes-Open Condition

Time-series analyses were performed for the eyes-open condition to compare the processed EOG and eye-tracking signals for each participant along the horizontal and vertical directions. For each participant, we used the calculated grand average of normalized signals across all eyes-open trials and compared the two modalities at each time point. Both the EOG and eye-tracking signals were downsampled to match the eye tracker’s frame rate of 30 Hz. Given the trial duration of 24 s, each trial consisted of 720 time-aligned samples. Before the statistical comparison, we assessed the normality of the paired signal differences using the Shapiro–Wilk test. If the normality assumption was met, we selected a parametric paired *t*-test. If not, we used a non-parametric Wilcoxon signed-rank test to perform the paired comparison tests. In addition, we applied two one-sided equivalence tests (TOSTs) to evaluate whether the differences between modalities were small enough to be considered practically negligible. The smallest effect size of interest (SESOI) was set to a Cohen’s d of 0.1, often interpreted as a trivially small effect according to widely used benchmarks [60,61]. We converted this SESOI into raw measurement units using the standard deviation of the paired differences, producing symmetric upper and lower equivalence bounds. The equivalence test assessed whether the observed mean differences (90% confidence interval (CI)) fell entirely within these bounds, indicating practical equivalence.

#### 4.4.2. Comparing EOG Signals and the Ground Truth Trajectory for the Eyes-Closed Condition

While eye tracking offers an alternative sensing modality for reference, direct comparisons with it are not feasible under closed-eye conditions. Therefore, we conducted a control analysis using a surrogate data approach, following the method described in [53] to evaluate whether the EOG signals accurately captured the target trajectories during eyes-closed conditions. Specifically, we compared the MAE between the true data (EOG signals aligned with their paired target trajectories) and the surrogate data (EOG signals aligned with shuffled target trajectories). The surrogate data were generated by randomly shuffling the trajectory sequence 100 times to avoid bias from any single random permutation. After checking for normality with Shapiro–Wilk tests, we applied either a paired *t*-test or a non-parametric Wilcoxon signed-rank test to determine the statistical significance of the MAE differences.

## 5. Results

### 5.1. Effect of Lighting Conditions on Similarity Between EOG and Eye-Tracking Signals Under Eyes-Open Conditions

**Figure 6:**
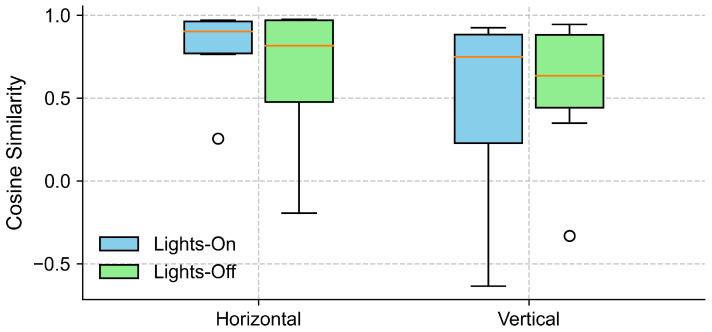
Cosine similarity between EOG and eye-tracking signals under different lighting conditions. The boxplots show the distribution of cosine similarity values across participants for each gaze direction (horizontal and vertical), comparing lights-on (blue) and lights-off (green) conditions. Each box represents the interquartile range (IQR), with the horizontal line indicating the median, and the whiskers extending to the minimum and maximum values within 1.5 × IQR. Circles represent outliers.

To examine the effect of lighting conditions on the alignment between the EOG and eye-tracking signals, we computed the cosine similarity between the grand-averaged EOG and eye-tracking signals separately for lights-on and lights-off conditions, in both the horizontal and vertical gaze directions (see Section 4.2). As shown in Figure 6, the horizontal direction yielded a high cosine similarity across the participants under both lighting conditions, with slightly higher and less variable values observed for the lights-on trials. In contrast, the cosine similarity in the vertical direction exhibited a greater variability.

**Figure 7:**
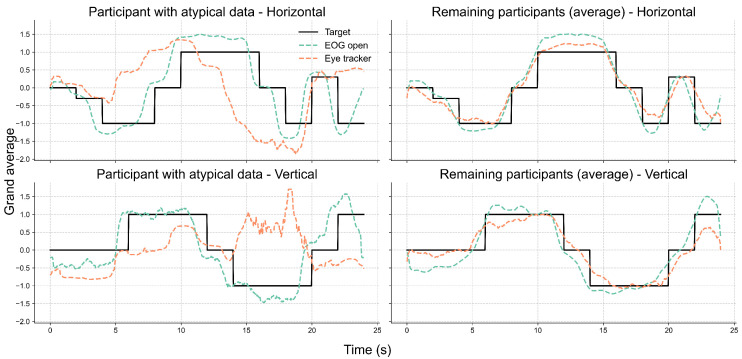
Comparison of eye movement signals between a participant with myopia and uncorrected vision and the group average. Each subplot displays the grand-averaged signals over time for the horizontal (**top row**) and vertical (**bottom row**) directions. The **left column** shows data from one participant whose eye-tracking signals exhibited low alignment with the EOG and target trajectories. The **right column** presents the average signals from the remaining participants, excluding that case. Black lines represent the target trajectory, while dashed lines indicate the EOG (green) and eye-tracker (orange) signals recorded under the eyes-open condition.

**Figure 8:**
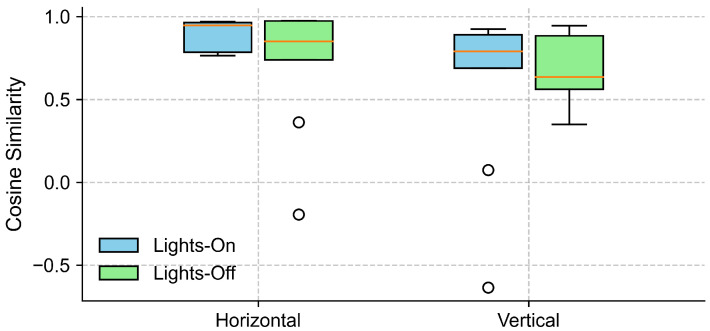
Cosine similarity between EOG and eye-tracking signals under different lighting conditions, excluding data from one participant with myopia (–6D corrective lens) and uncorrected vision. The boxplots show the distribution of cosine similarity values across participants for each gaze direction (horizontal and vertical), comparing lights-on (blue) and lights-off (green) conditions. Each box represents the interquartile range (IQR), with the horizontal line indicating the median, and the whiskers extending to the minimum and maximum values within 1.5 × IQR. Circles represent outliers.

Further inspection revealed that the extreme outliers were primarily attributable to the data from one participant. This participant had myopia requiring –6D corrective lenses and was not wearing their glasses during the experiment. Figure 7 illustrates the EOG and eye-tracking signals during the eyes-open trials for the participant with reduced signal alignment and the average signals from the remaining participants. It is evident that the eye-tracking signals from this specific participant demonstrated poor alignment with both the target trajectory and the corresponding EOG signals. In contrast, the EOG signals were unaffected by the participant’s visual disorder and closely followed the target trajectory. Therefore, we recalculated the cosine similarity after excluding this participant. The updated results in Figure 8 show a clear reduction in variability across all conditions, particularly in the vertical direction under the lights-on condition. We summarized the statistical test results that assessed the effect of lighting conditions in Table 2. We first tested for normality using the Shapiro–Wilk test for cosine similarity values under the “lights-on” and “lights-off” conditions. Most of the conditions showed significant deviations from normality (p<0.05), except for the vertical “lights-off” condition (p=0.199). Based on these results, we employed the non-parametric Wilcoxon signed-rank test to compare the similarity scores between the lighting conditions. No significant differences were found in either the horizontal (W=16, p=0.496) or vertical (W=19, p=0.734) directions. The corresponding effect sizes were small to moderate (d=0.45 for horizontal, d=−0.25 for vertical).

### 5.2. Grand Average of Instructed Trajectory vs. Sensed Gaze Across Lighting Conditions

**Figure 9:**
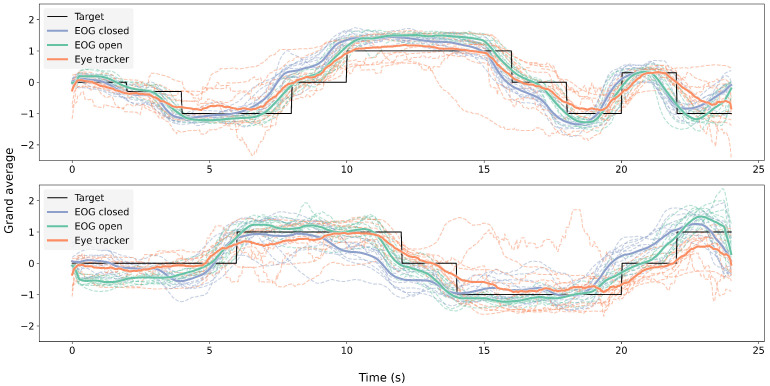
Grand average per participant of EOG with open (green) and closed (blue) eyes and eye-tracking signals (orange) with open eyes. The upper graph shows horizontal eye movement, while the bottom graph shows vertical eye movement.

Figure 9 shows the grand average of EOG signals during the eyes-open and eyes-closed trials, the eye-tracking signals during the eyes-open trials, and the corresponding target trajectories in both the horizontal and vertical directions across 11 participants.

### 5.3. Cross-Correlation Between Instructed Trajectory and Sensed Gaze

**Figure 10:**
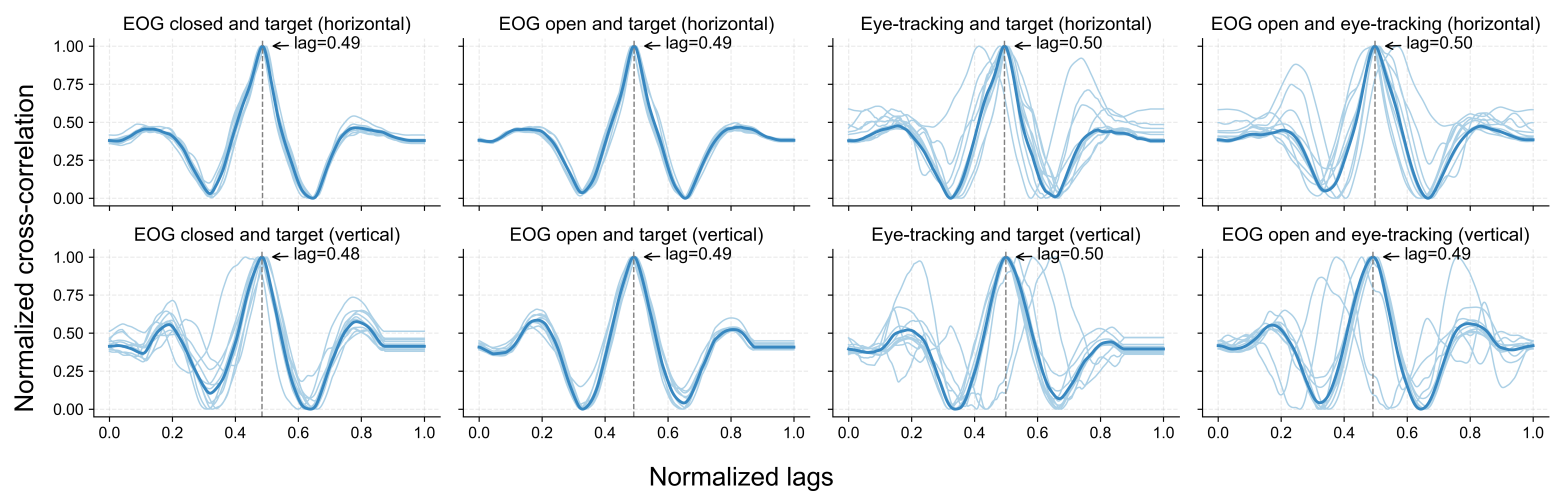
Normalized cross-correlation between eye movement signals and target trajectories. Each subplot shows the normalized cross-correlation between signal pairs or between a signal and the target trajectory, including EOG under eyes-open and eyes-closed conditions, eye-tracking data under eyes-open conditions, and target trajectories, in both horizontal and vertical directions. Light blue lines represent individual participants (N = 11), while the dark blue line indicates the group-level results. Dashed vertical lines mark the lag at which the maximum correlation occurs.

Figure 10 illustrates the cross-correlation between pairs of signals or between a signal and the target trajectory across the horizontal and vertical directions. After scaling the cross-correlation index to the [0, 1] range, a peak correlation of around 0.5 indicates that the highest correlation between two signals occurred when they were temporally aligned. In other words, a peak in 0.5 indicates that there is no lag between the signals. Across all the comparisons, the group-level cross-correlation curves exhibited a clear peak near the normalized lag of 0.5. In the horizontal direction, the average delay between the EOG and eye-tracking signals was approximately 50 ms, with the EOG preceding the eye-tracking curve. In the vertical direction, this delay was around 200 ms, again indicating that EOG preceded the eye-tracking signals.

### 5.4. Comparison of EOG and Eye-Tracking Signals Under Eyes-Open Conditions

Normality tests with the Shapiro–Wilk test indicated a deviation from a normal distribution (p<0.05). Therefore, we employed non-parametric Wilcoxon tests instead of paired *t*-tests. The Bonferroni correction was used to correct for multiple comparisons.

Table 3 summarizes the results of the Wilcoxon signed-rank tests and equivalence tests for the horizontal and vertical gaze components under eyes-open conditions. The test statistics and equivalence outcomes for each individual participant are reported. Additionally, the *All* row indicates the group-level analysis based on the average signals across all participants.

At the group level, no statistically significant differences were observed between the EOG and eye-tracking signals for horizontal (p=1.000) or vertical (p=1.000) movements. Additionally, the 90% confidence intervals of the mean differences ([−0.019, 0.019] for horizontal; [−0.027, 0.027] for vertical) fell entirely within the predefined equivalence bounds (±0.013 and ±0.043, respectively), thereby confirming statistical equivalence. This pattern was consistent across all the participants, where all *p*-values exceeded 0.05 and all 90% confidence intervals remained within the participant-specific equivalence bounds. Even in cases with relatively larger bounds (e.g., P01 and P05), the equivalence criteria were still met. These results provide strong evidence that EOG and eye-tracking signals are not only statistically indistinguishable, but also statistically equivalent across both spatial dimensions.

### 5.5. Control Analysis of EOG Accuracy Under Eyes-Closed Conditions Using Surrogate Data

**Figure 11:**
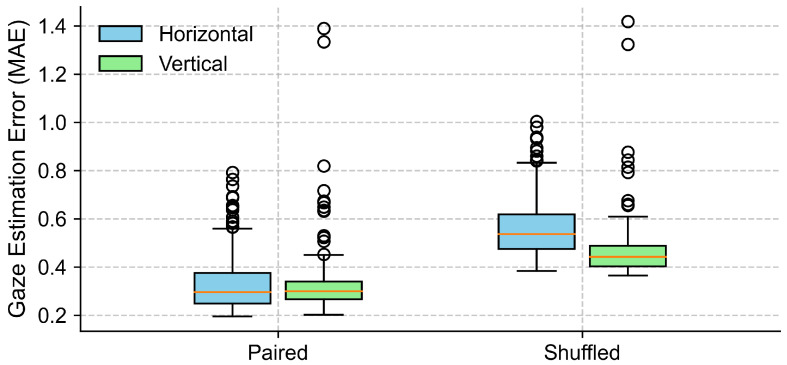
Comparison of gaze estimation errors (MAE) between true (paired) and surrogate (shuffled) data for both horizontal and vertical directions. Each box represents the distribution of trial-level mean absolute errors (MAEs), with lower values indicating better alignment between EOG signals and target trajectories. The boxes represent the interquartile range (IQR), with the horizontal line indicating the median, and the whiskers extending to the minimum and maximum values within 1.5 × IQR. Circles represent outliers.

To evaluate the effectiveness of EOG signals in capturing gaze trajectories under eyes-closed conditions, we compared the MAE between the true data and surrogate data across the horizontal and vertical directions. As shown in Figure 11 and Table 4, the paired condition yielded lower MAEs compared to the shuffled condition. In the horizontal direction, the average MAE increased from 0.334±0.123 in the paired condition to 0.565±0.128 in the shuffled condition. Normality testing using the Shapiro–Wilk test indicated a violation of the normality assumption (*p* < 0.001), so a non-parametric Wilcoxon signed-rank test was applied. This test confirmed a statistically significant difference between the conditions (*p* < 0.001), with a large effect size (Cohen’s d=4.58). Similarly, in the vertical direction, the average MAE rose from 0.327±0.135 to 0.465±0.120. The normality assumption was violated (Shapiro–Wilk *p* < 0.001), and the Wilcoxon signed-rank test confirmed the difference as statistically significant (*p* < 0.001), with an effect size of Cohen’s d=1.85. These results suggest that EOG signals contain meaningful information about the eye movement direction during eye closure, and that the gaze direction estimated from EOG was significantly more accurate than that derived from shuffled control data, which reflects the chance-level performance.

## 6. Discussion

We designed an experiment to evaluate gaze direction estimation with EOG and an eye tracker under four conditions: lights on and eyes open, lights on and eyes closed, lights off and eyes open, and lights off and eyes closed. A gaze direction was instructed using audio and proprioceptive cues. With these data, we assessed the agreement between the gaze direction estimates from EOG and video-based eye tracking. Our results demonstrated a strong alignment between EOG-based and eye-tracking-based gaze signals in both the horizontal and vertical directions when the participants’ eyes were open. We compared the similarity between the EOG and eye-tracking signals under both lights-on and lights-off conditions to understand whether ambient lighting influenced gaze measurements. The results showed a comparable performance across both conditions. In addition, we investigated whether detecting the eye movement direction using EOG under the eyes-closed condition is possible. During the eyes-closed trials, the EOG signals retained meaningful directional trends that followed the target trajectory that the participants were instructed to follow. Our statistical analyses also confirmed that the EOG-based gaze estimates were significantly more accurate than chance.

In the literature, ambient light is recognized as a confounding factor for eye tracking, and, to a lesser extent, for EOG as well. However, our results showed that the similarity between the EOG and eye-tracking signals remained high and comparable under both lights-on and lights-off conditions (see Figure 8). The statistical results further showed no significant differences between lighting conditions in either the horizontal or vertical direction, indicating that ambient illumination had no measurable impact on the signal alignment (see Table 2). The eye-tracking system demonstrated a stable performance regardless of lighting, likely due to its use of an infrared (IR) illuminator that enables robust tracking, even in the absence of visible light. A particular case involved one participant suffering from myopia who exhibited consistently lower similarity values across both lighting conditions along the two directions. This discrepancy was likely due to frequent squinting caused by refractive errors. Squinting could have led to the partial occlusion of the eyes and, therefore, hindered the performance of video-based eye tracking. Nevertheless, the EOG signals from this participant remained well aligned with the target trajectory, which highlights the usability of EOG under such challenging conditions.

The grand-average signals from both modalities closely followed the target trajectory over time across both lighting conditions, as illustrated in Figure 9, with some deviation across participants. The cross-correlation analysis (Figure 10) further supported this finding, revealing consistent correlation peaks near a normalized lag of 0.5 across all signal pairs. Notably, the EOG signals tended to slightly precede the eye-tracking signals, which may reflect the earlier detection of oculomotor activity via EOG compared to the visual-capture latency of eye trackers. Similar observations of electromyography (EMG) signals preceding computer vision detection have been reported in prior studies [62,63].

Furthermore, we found EOG tracking to be equivalent to eye tracking when the eyes were open, and to the instructed gaze trajectory with the eyes closed, as indicated by the Wilcoxon signed-rank tests and two one-sided tests (TOSTs) in both the horizontal and vertical directions. Looking closer, the alignment between EOG and eye tracking was stronger in the horizontal direction than in the vertical. This was reflected in the higher similarity and tighter overlap between the horizontal EOG and eye-tracking signals (Figure 8 and Figure 9), the sharper and more consistent cross-correlation peaks (Figure 10), and the smaller MAE and larger effect size observed for the horizontal axis during the gaze estimation error analysis (Table 4). This finding aligns with previous studies, which noted that vertical EOG signals were often too noisy and heavily influenced by horizontal ocular activities to extract useful vertical eye movement features [54,55]. The reduced reliability of vertical EOG signals may be explained by their greater susceptibility to noise and artifacts from involuntary actions such as blinking and squinting.

With eyes closed, the gaze detected from the EOG signals preserved a direction that was qualitatively consistent with the target trajectory, as shown in Figure 9. Our quantitative analysis (Figure 11, Table 4) further confirmed that the EOG-based gaze estimates were significantly more accurate than those derived from shuffled (chance-level) data, for both the horizontal and vertical components. The large effect sizes observed underscore the robustness of this effect. To further assess the adequacy of our sample size, we conducted a post hoc power analysis based on the observed effect sizes (Cohen’s d = 4.58 for horizontal and d = 1.85 for vertical error comparisons). With a sample size of 11 participants and a significance level of alpha = 0.05 (two-tailed), the statistical power for both comparisons exceeded 0.99. These results indicate that our study had sufficient power to detect the observed effects.

Together, our findings support the feasibility of using EOG as a viable alternative to video-based eye tracking under standard conditions, where the latter may suffer from a reduced accuracy due to eyelid interference or blinking, as we observed in one myopic participant with a decreased performance. Furthermore, both video-based and conventional EOG-based gaze estimation methods typically require a calibration procedure that relies on visual fixation and is therefore limited to open-eye conditions. In contrast, the proposed proprioceptive calibration method enables EOG-based gaze estimation without requiring visual input, making it a promising standalone tool for use with the eyes closed or in situations where visual input is unavailable. This is especially important in investigating the relationship between eye movement and sensory processes other than vision. For example, it could be used to investigate eye movement while closing the eyes and listening to sound stimuli during sleep research, and to measure the eye movements of blind individuals. Enabling eyes-closed tracking could serve as the basis to uncover physiological mechanisms for automatic eye movement navigation, such as head and gaze anticipation in both blind and sighted people [31]. Other applications of measuring the EOG with the eyes closed include detecting involuntary eye movements during closed-eye periods [64], which would allow sleep quality assessments or the early detection of diseases, for example, Parkinson’s disease [65,66]. Moreover, in interaction designs, blind people do not use their eyes for vision anymore, freeing an interaction channel with voluntary movement control. This new channel would be extremely useful if the hands were used as tactile sensory substitution devices, becoming unable to freely interact with and manipulate objects. Given its simplicity, cost-effectiveness, and robustness to lighting and occlusion, EOG could be integrated into mobile or wearable systems where camera-based solutions are less practical.

## 7. Limitations and Future Work

The proprioceptive calibration method proposed in this study provides a practical solution for estimating the gaze direction in scenarios where visual input is unavailable or the eyes are closed. Unlike common calibration techniques that depend on visual fixation, this method derives user-specific parameters to interpret EOG signals by guiding users through tactile targets and auditory instructions. However, even for identical eye movements, the resulting EOG signals can vary substantially across individuals and recording sessions due to anatomical differences and electrode placement variability. Therefore, while effective within individual sessions, a calibration must be performed to ensure a consistent accuracy in new sessions or with different users. Developing robust methods that can generalize across users and sessions without requiring repeated calibration is a valuable and challenging direction for future research. In particular, future work could explore techniques to compensate for variations in electrode placement or model user-specific signal characteristics. Additionally, it would be worthwhile to compare the proposed proprioceptive calibration with alternative methods suitable for closed-eye conditions, such as those based on verbal instructions. In this study, we prioritized multiple short-duration calibration trials and did not evaluate the long-term robustness of the method in continuous, single-session recordings. While the temporal robustness of EOG-based gaze estimation has been well studied under open-eye conditions, such an analysis has yet to be extended to closed-eye scenarios. Future work should investigate the stability of EOG signals and the performance of the gaze estimation method over extended recording periods under eyes-closed conditions, to assess its reliability in extended-use applications such as sleep monitoring or assistive interfaces.

Our results revealed that vertical gaze direction estimations tend to be noisier and less reliable than horizontal ones (Figure 8, Figure 9 and Figure 10). This imbalance was reflected in a weaker alignment with CV-based gaze estimates and larger errors along the vertical axis. Future work should explore signal-processing techniques or sensor configurations that can improve the quality of vertical EOG signals to enhance the robustness of full 2D gaze estimation. Moreover, the current study primarily focused on offline signal processing and statistical validation to demonstrate the feasibility of the proposed method. Extending this work to support real-time closed-eye gaze estimation represents a critical next step toward enabling practical, interactive applications.

Another limitation of this study is that the evaluations were conducted in the laboratory. While this was important for validating the core method under consistent conditions, it restricted insight into the method’s real-world applicability, particularly in dynamic or mobile environments such as wearable or assistive technologies. Future work should evaluate the sensing performance in real-world settings to assess the practical usability in gaze-based interaction applications. Additionally, we observed a notable drop in performance in one myopic participant’s CV-based eye-tracking data, while their EOG signals remained stable and of good quality. This case highlights the need to further investigate the system performance across a broader range of user profiles and visual acuity. Future work should aim to include a more diverse participant pool to better capture inter-individual variability and strengthen the external validity of the findings. Investigating user-specific factors that affect signal quality and system performance, such as anatomical variation, ocular conditions, and the use of corrective lenses during EOG recording, will be essential for developing more generalizable and user-adaptive EOG-based gaze estimation systems.

## Figures and Tables

**Table 1:** Comparison of EOG- and CV-based eye tracking.

Criteria	EOG	Eye Tracking
**Measurements** [26]	Measures the corneoretinal standing potential using electrodes around the eyes	Analyzes infrared light reflected from the eye using video-based cameras
**Occlusion** [13,26]	**Can track under eyelids**	Cannot track when eyes are closed or occluded
**Illumination sensitivity** [14,15,16]	Ambient lighting might cause small drifts [25]	Performance may degrade in bright outdoor environments or in dark conditions without infrared illumination
**Optical interface sensitivity** [17]	**Not affected by reflections**	Performance may degrade due to optical interference from ambient infrared light and reflective surfaces
**Spatial accuracy** [27,28]	Lower (approx. 1–2° error)	**Higher (up to 0.5° or better)**
**Temporal resolution** [27,29]	**Higher (up to 1 kHz)**	Lower (typically 60–300 Hz; high-end systems can reach up to 1000 Hz)
**Computational load** [26]	**Lower (signal processing)**	Higher (video acquisition and image analysis)

Note: Bold text indicates the method with better performance for the given criterion. If neither method is highlighted, it means both have comparable limitations or no clear advantage.

**Table 2:** Normality (Shapiro–Wilk) and Wilcoxon signed-rank test results for cosine similarity between lights-on and lights-off conditions.

Direction	Condition	Shapiro–Wilk (*W*, *p*-Value)	Wilcoxon (*W*, *p*-Value)	Cohen’s *d*
Horizontal	Lights on	0.775, 0.011	16, 0.496	0.45
	Lights off	0.731, 0.003		
Vertical	Lights on	0.699, 0.001	19, 0.734	−0.25
	Lights off	0.889, 0.199		

**Table 3:** Summary of Wilcoxon signed-rank tests and equivalence tests (TOSTs) comparing EOG and eye-tracking signals for both the horizontal (X) and vertical (Y) gaze directions under eyes-open conditions, including both individual and group-level results.

Participant	*W*-Stat X	*p*-Val X	Equiv Bound X	90% CI X	Equiv X	*W*-Stat Y	*p*-Val Y	Equiv Bound Y	90% CI Y	Equiv Y
All	124,940	1.000	±0.031	±0.019	Yes	124,130	1.000	±0.043	±0.027	Yes
P01	125,160	1.000	±0.110	±0.068	Yes	116,194	0.359	±0.119	±0.073	Yes
P02	117,783	0.759	±0.023	±0.014	Yes	121,977	1.000	±0.038	±0.023	Yes
P03	121,785	1.000	±0.071	±0.044	Yes	124,363	1.000	±0.053	±0.032	Yes
P04	118,439	1.000	±0.023	±0.014	Yes	129,296	1.000	±0.036	±0.022	Yes
P05	120,971	1.000	±0.055	±0.034	Yes	128,942	1.000	±0.121	±0.074	Yes
P06	120,104	1.000	±0.022	±0.013	Yes	127,549	1.000	±0.044	±0.027	Yes
P07	117,370	0.629	±0.041	±0.025	Yes	127,024	1.000	±0.047	±0.029	Yes
P08	129,136	1.000	±0.060	±0.037	Yes	121,666	1.000	±0.090	±0.055	Yes
P09	128,758	1.000	±0.052	±0.032	Yes	115,432	0.244	±0.049	±0.030	Yes
P10	123,344	1.000	±0.021	±0.013	Yes	123,664	1.000	±0.045	±0.028	Yes
P11	128,490	1.000	±0.071	±0.044	Yes	124,597	1.000	±0.061	±0.037	Yes

**Table 4:** Comparison of gaze estimation error (MAE) between true (paired) and surrogate (shuffled) data for *X* and *Y* axes.

Axis	Condition	MAE (Mean ± SD)	Shapiro–Wilk (*W*, *p*-Value)	Wilcoxon (*W*, *p*-Value)	Cohen’s *d*
X	Paired	0.334 ± 0.123	0.828, <0.001	0, <0.001	−4.58
	Shuffled	0.565 ± 0.128	0.904, <0.001		
Y	Paired	0.327 ± 0.135	0.536, <0.001	107, <0.001	−1.85
	Shuffled	0.465 ± 0.120	0.582, <0.001		

## Data Availability

The original data presented in the study are openly available in OSF: https://doi.org/10.17605/OSF.IO/PTVKQ (accessed on 26 October 2025).
